# Fluoroscopic guidance for bulla identification during ventral bulla osteotomy in eight French bulldogs

**DOI:** 10.1002/vro2.70008

**Published:** 2025-04-10

**Authors:** Hui Yu Lu, Jeffery J. Biskup, Lea R. Mehrkens, Debbie Reynolds

**Affiliations:** ^1^ Toronto Animal Health Partners Specialty and Emergency Hospital North York Ontario Canada; ^2^ Capital City Specialty and Emergency Animal Hospital Kanata Ontario Canada

**Keywords:** canine, fluoroscopic‐guided, french bulldogs, otitis media, ventral bulla osteotomy

## Abstract

**Objective:**

To describe the technique and outcome of fluoroscopy to guide bulla identification in French bulldogs during ventral bulla osteotomy.

**Materials and methods:**

Medical records of eight French bulldogs with otitis media that underwent a fluoroscopic‐guided ventral bulla osteotomy between January 2020 and June 2023 were reviewed. Demographics, preoperative diagnostic findings, advanced imaging findings, surgical times, histopathology and culture results, as well as postoperative outcomes, were recorded.

**Results:**

Following routine dissection of the bulla, fluoroscopic imaging was used to confirm the placement of a Steinmann pin before bulla penetration. The median surgical time was 152 minutes (range: 80–210 minutes). All dogs survived to discharge. Six out of eight dogs retained an ipsilateral head tilt postoperatively. Two dogs exhibited residual vestibular ataxia at 14 days postoperatively, which improved and resolved 7 months and 2 years postoperatively respectively. One dog developed recurring otitis media, and a total ear canal ablation and lateral bulla osteotomy were recommended.

**Clinical significance:**

Intraoperative fluoroscopy can be used successfully to guide the identification of the bulla in ventral bulla osteotomies in brachycephalic breeds.

## INTRODUCTION

Brachycephalic dog breeds’ unique skull conformation predisposes to several disease processes, with several studies reporting associations between brachycephaly and middle ear disease because skull conformation obstructs the Eustachian tube.^1^, ^2^, ^3^, ^4^ In 55 brachycephalic dogs that had computed tomography (CT) for respiratory signs, middle ear effusion was discovered incidentally in 53% of French bulldogs.^5^ Similarly, in a study of routine skull CT imaging of 75 French bulldogs and pugs undergoing brachycephalic airway corrective surgery, 40% of dogs exhibited middle ear effusion and 82% had narrowed ear canals.^6^ It has been hypothesised that compromise to the patency of the Eustachian tubes results in a buildup of ceruminous debris and fluid in the middle ear, creating an ideal microenvironment for the proliferation of bacteria and subsequently leading to otitis media.^7^

In cases where medical management for otitis media does not improve clinical signs or there is a central extension of disease, ventral bulla osteotomy (VBO) is a viable surgical treatment option to remove debris and diseased tissue of the middle ear and to debride the bulla lining while leaving the external canal intact in patients that do not have a history of otitis externa.^8^, ^9^, ^10^ Ventral bulla osteotomy is an alternative treatment option to a total ear canal ablation and lateral bulla osteotomy (TECA‐LBO) as it has the potential to preserve any residual hearing ability.^11^ Identification of the bulla is challenging in brachycephalic dogs due to their unique skull conformation, small bulla size, and thick muscle overlying the bulla.^12^ Intraoperative image guidance has been reported to allow for better identification of the bulla in dogs receiving a VBO for cholesteatoma. This technique has only been reported in dolichocephalic dogs.^8^ Fluoroscopy is an intraoperative imaging modality typically available in referral veterinary hospitals due to its frequent use in fracture repairs.^13^ To the best of the authors' knowledge, there are no studies describing the use of intraoperative fluoroscopy to identify the bulla.

The objective of this study was to describe the technique and outcomes of intraoperative fluoroscopy in bulla identification for VBOs in French bulldogs with otitis media.

## MATERIALS AND METHODS

Medical records for French bulldogs that underwent a fluoroscopic guided VBO at Toronto Animal Health Partners Emergency and Specialty Hospital between 1 January 2020 and 1 June 2023 were reviewed and enrolled. Data collected included signalment, weight, laterality (left, right or bilateral), surgical indication (primary otitis media or secondary otitis media due to cholesteatoma or other tumours), physical examination findings, preoperative bloodwork, preoperative imaging (magnetic resonance imaging [MRI] and/or CT) findings, cerebrospinal fluid (CSF) findings, duration of surgery, postoperative CT findings, intra‐ and postoperative complications and histopathology results. The vertical distance from the ventral bulla to the skin was measured in the preoperative CT images using the Keystone Omni software (Asteris).

All dogs received a 14‐day postoperative recheck involving history collection and a physical examination. Subsequent follow‐up, more than 14 days postoperatively, was performed either by a telephone call or an in‐person recheck comprising history collection and a physical examination.

### Surgical technique—Fluoroscopic guided VBO

The dogs were placed under general anaesthesia and were positioned in ventral recumbency for CT and MRI scans. All dogs were positioned in dorsal recumbency for surgery, with the ventral neck clipped and aseptically prepared. A rolled towel was placed under the dorsal cervical region to elevate the caudal skull.

**FIGURE 1:**
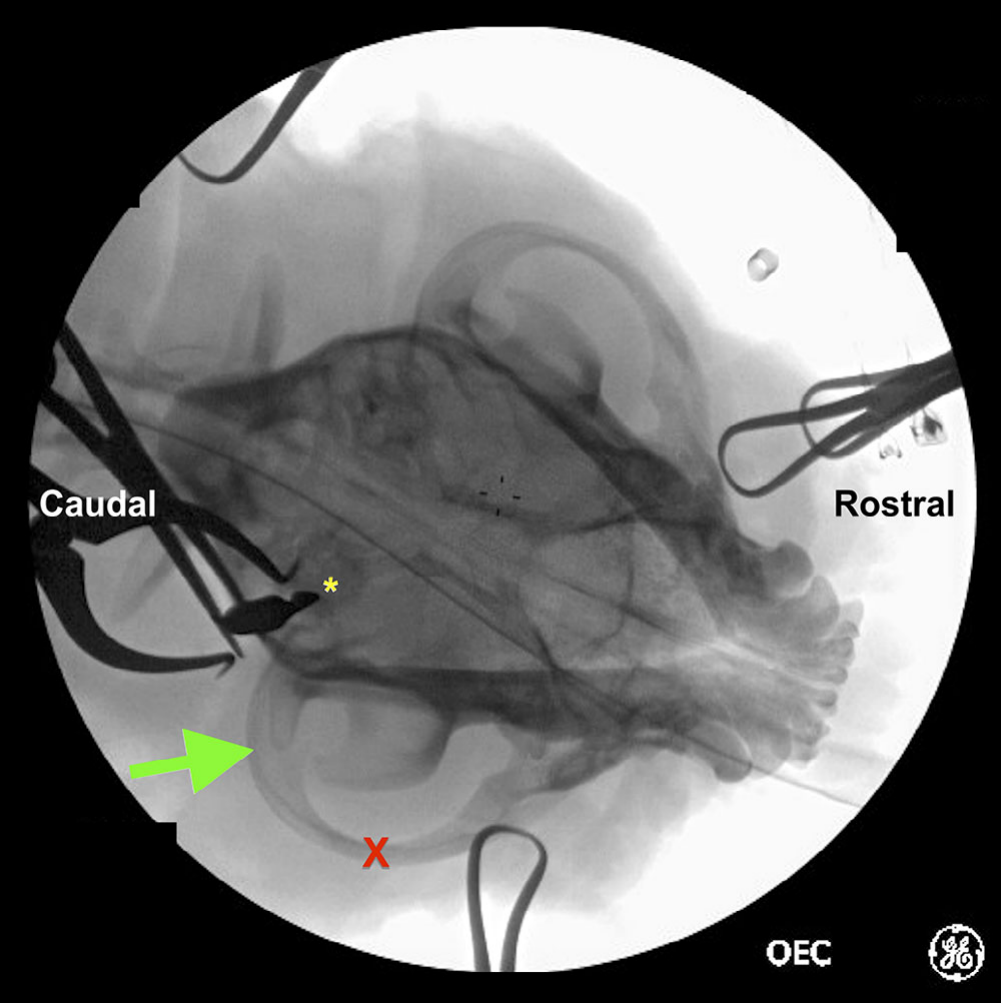
Ventrodorsal fluoroscopic image of French bulldog #8 undergoing fluoroscopic guided ventral bulla osteotomy. Following soft tissue dissection and using fluoroscopic guidance, a Freer periosteal elevator was positioned over the ventral bulla (yellow asterisk) once identified. The zygomatic arch (red X) and vertical ramus of the mandible (green arrow) are as labelled.

**FIGURE 2:**
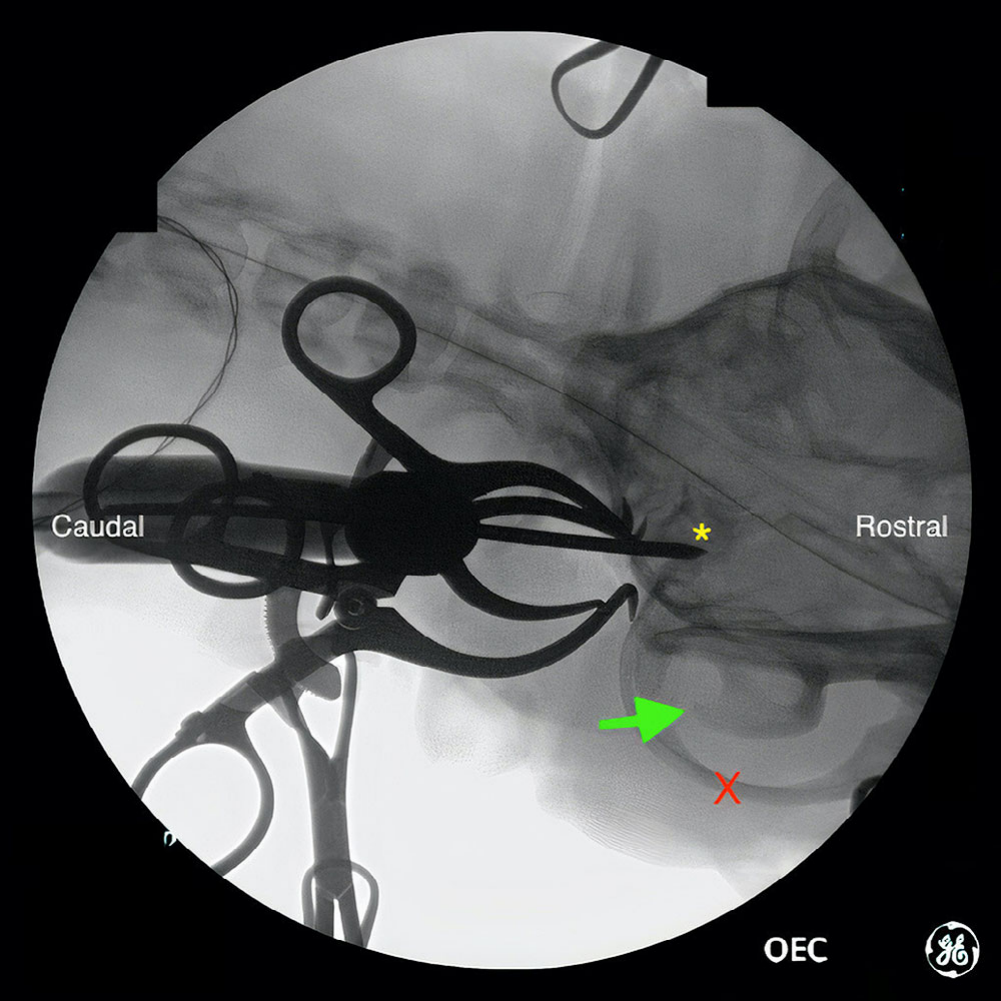
Ventrodorsal fluoroscopic image of French bulldog #8 undergoing fluoroscopic guided ventral bulla osteotomy. Following soft tissue dissection and using fluoroscopic guidance, a 3/32” Steinmann pin was advanced in the ventral bulla (yellow asterisk). The zygomatic arch (red X) and vertical ramus of the mandible (green arrow) are as labelled.

**FIGURE 3:**
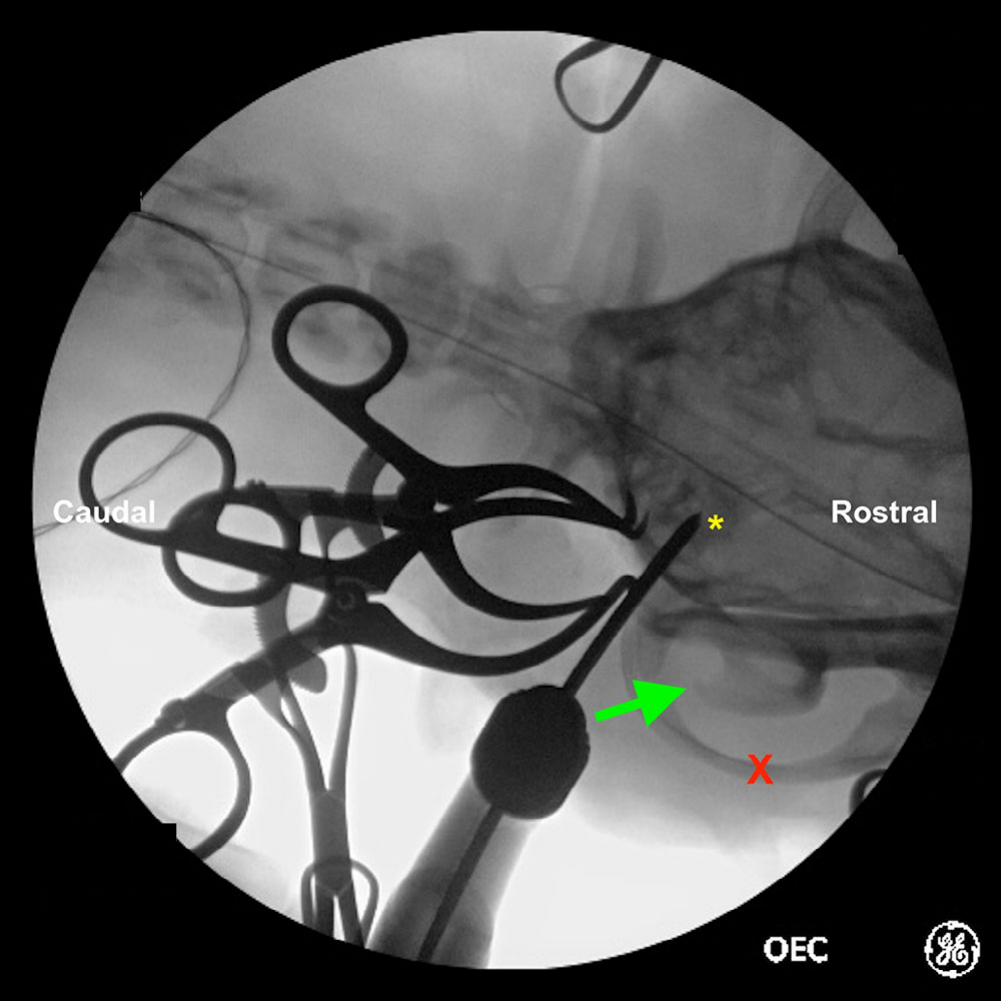
A second intraoperative ventrodorsal fluoroscopic image of French bulldog #8 undergoing fluoroscopic guided ventral bulla osteotomy. The ventral bulla (yellow asterisk), zygomatic arch (red X) and vertical ramus of the mandible (green arrow) are as labelled.

Fluoroscopic imaging was performed to allow identification of the bulla before skin incision. On the ventrodorsal view of the skull, the ventral bulla was identified as a circular radio‐opaque structure caudomedial to the zygomatic arch and vertical ramus of the mandible, as labelled in Figures 1, 2, 3.

A paramedian skin incision approximately 3 cm long was made over the region of the bulla. The subcutaneous tissues and the platysma muscles were bluntly dissected. The bifurcation of the linguofacial and maxillary veins was retracted laterally. The muscles (digastricus, mylohyoid, hyoglossus and styloglossus) ventral to the area of the bulla, medial to the vertical ramus of the mandible and ventral to the trachea were bluntly separated to allow exposure of the ventral aspect of the skull. Drop‐handled Gelpi retractors were used to maintain exposure. Once the bulla was identified, a 3/32‘‘ Steinmann pin was aimed ventrodorsally in the region of the bulla, and the pin was subsequently advanced into the bulla once the location was confirmed by fluoroscopy. The defect was enlarged with Kerrison rongeurs. The interior of the bulla was examined, and the bulla lining was removed using curettage with samples obtained for culture and sensitivity (*n* = 8/8) and histopathological examination (*n* = 7/8). The bulla was flushed with sterile saline and visually inspected to ensure that the bulla epithelium had been removed.

The deeper subcutaneous tissue was closed with a simple continuous pattern with polydioxanone 2‐0 suture (PDS II, Ethicon, Johnson and Johnson). The subcutaneous tissue and skin were closed using a simple continuous pattern and intradermal pattern, respectively, with poliglecaprone 25 3‐0 suture (Monocryl, Ethicon).

## RESULTS

### Signalment and presenting clinical signs

Eight French bulldogs met the inclusion criteria (Table 1). The median age was 3 years (range: 9 months to 11 years) and the median weight was 13.1 kg (range: 9.6–15.6 kg). Neutered male dogs (*n* = 5/8) were the most common, followed by spayed females (*n* = 2/8) and entire males (*n* = 1/8).

**TABLE 1:** Summary data of signalment, weight, clinical presentation findings, preoperative diagnostic results, duration of hospitalisation, anaesthesia and surgery, and vertical distance measurements in eight French bulldogs that underwent a fluoroscopic‐guided ventral bulla osteotomy.

Dog	Sex	Age (years)	Weight (kg)	Clinically affected side	Duration of clinical signs (days)	Preoperative bloodwork abnormalities	Abnormal side(s) on preoperative MRI imaging	CNS extension	CSF findings	Duration of anaesthesia (min)	Duration of surgery (min)	Duration of hospitalisation (days)	Vertical distance from skin to ventral bulla (mm)
1	FS	3	11.8	Left	10	NA	Bilateral	Yes	Protein 162.7 mg/dL WBC 5304/µL	165	90	4	56.9
2	MN	0.75	9.6	Right	35	ALT 147 U/L	Bilateral	No	NA	240	105	2	NA
3	MN	5	15.8	Left	60	Neutrophilia 13 × 10^9^/L	Left	No	NA	240	195	1	NA
4	MN	3	14.4	Right	5	Hyperbilirubinemia 17 µmol/L	Right	Yes	Protein 647.2 mg/dL WBC 1664/µL	195	180	2	56.8
5	FS	3	10.5	Left	5	NA	Left	Yes	Protein 200 mg/dL WBC 217.6/µL	305	210	1	56.3
6	ME	2.5	15.6	Left	2	Leukocytosis 18 × 10^9^/L	Left	Yes	Protein 23 mg/dL WBC 92.8 /µL	180	135	2	41.3
7	MN	4	11	Right	4	Neutrophilia 12.6 × 10^9^/L, ALT 287 U/L	Bilateral	No	NA	260	170	2	45.0
8	MN	11	15.4	Right	334	Hyperglobulinemia 47 g/L	Right	Yes	Protein 20 mg/dL WBC 0/µL	170	80	2	70.4

Abbreviations: ALT, alanine aminotransferase; CNS, central nervous system; CSF, cerebrospinal fluid; FS, female spayed; MN, male neutered; ME, male entire; MRI, magnetic resonance imaging; NA, not applicable; WBC, white blood cell count.

All dogs presented with signs of a head tilt ipsilateral to the clinically affected ear, with four of eight dogs exhibiting a left‐sided head tilt and four exhibiting a right‐sided head tilt. On otoscopic examination, severe stenosis of the external ear canals was not appreciated in any of the eight dogs. Other reported clinical signs included vestibular ataxia (*n* = 6/8), nystagmus (*n* = 4/8), unilateral facial nerve paralysis (*n* = 2/8), inappetence (*n* = 3/8), unilateral aural discharge (*n* = 1/8), lethargy (*n* = 1/8), pain on opening mouth (*n* = 1/8) and restlessness (*n* = 1/8).

### Duration of clinical signs and preoperative medical treatment

The median duration of clinical signs before evaluation was 7.5 days (range: 2–334 days) (Table 1). Six dogs were receiving systemic oral antibiotics before surgery. Three dogs (*n* = 3/6) received amoxicillin–clavulanate acid, and the remaining dogs received clindamycin, metronidazole and enrofloxacin, respectively.

### Preoperative diagnostic testing

All dogs had preoperative serum biochemistry and complete blood count, with three dogs exhibiting an inflammatory leukocytosis characterised by mild neutrophilia (12.6 × 10^9^/L and 13.0 × 10^9^/L, respectively, range: 2.9–11.6 × 10^9^/L). Two dogs displayed a mildly elevated alanine aminotransferase (147 and 246 U/L, respectively; range: 10–125 U/L), one dog exhibited hyperglobulinemia (47 g/L; range: 25–45 g/L) and one dog exhibited a mild elevation in total bilirubin (17 µmol/L; range: 0–15 µmol/L) (Table 1).

**FIGURE 4:**
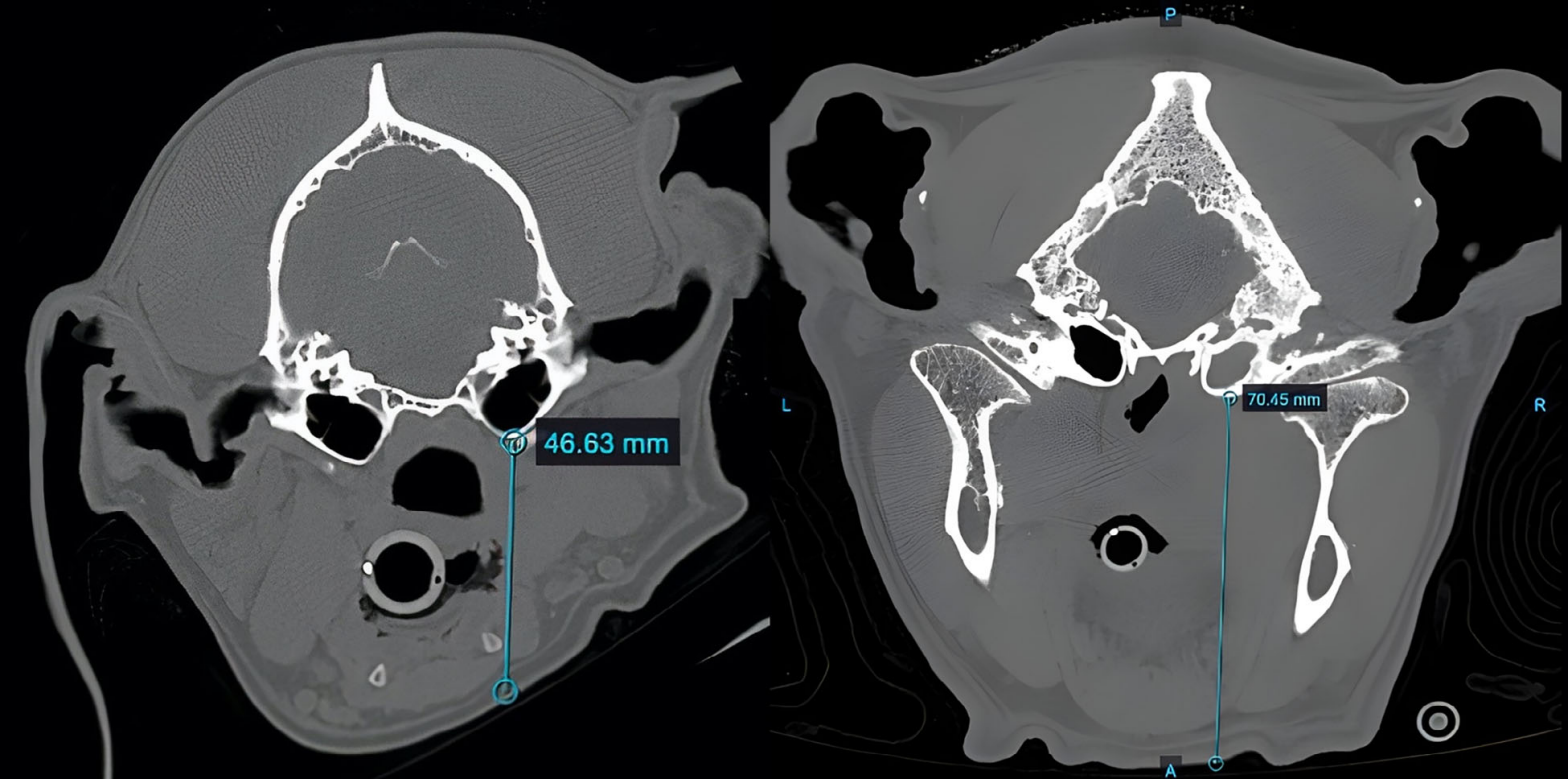
Axial preoperative computed tomography image of a brachycephalic dog (French bulldog #8) skull with the distance from the ventral bulla to skin measured at 70.4 mm, respectively, using the Keystone Omni software (asterisk).

All dogs underwent a head MRI scan preoperatively (Table 1). Homogenous and isointense T2 hyperintensity was observed in both bullae in three of eight dogs and the clinically affected bulla in the remaining five of eight dogs. Contrast enhancement of the muscles surrounding the affected bulla was observed in all dogs. Five dogs exhibited concurrent signs of extension into the cerebrum on the clinically affected side. There was a mildly compressive cervical intervertebral disc extrusion in one dog. Six of eight dogs underwent a preoperative CT to assist in surgical planning. Based on the preoperative CT images, the median vertical distance from the skin of the ventral mandible to the ventral bulla was 56.6 mm (range: 41.3–70.4 mm) (Figure 4).

A CSF tap from the cerebellomedullary cistern was performed in the five dogs that were suspected of exhibiting signs of cerebrum extension based on MRI imaging. The median protein level of the CSF fluid was 191 mg/dL (range: 20–647 mg/dL; reference range: <30 mg/dL) and the median leukocyte count was 217/µL (range: 0–5304/µL, reference range: 0–4/µL). No atypical neoplastic cells or infectious organisms were identified in any of the five samples. The aerobic and anaerobic cultures of the CSF fluid were negative for growth in all dogs.

### Intraoperative complications

Three surgeons performed the surgery on eight dogs with identification of the bulla successfully achieved, with two to three adjustments in pin placement before entry into the bulla. No significant intraoperative haemorrhage was encountered. Intraoperative damage to the nerves around the bulla was not identified postoperatively. All eight dogs recovered uneventfully from anaesthesia aside from one dog exhibiting drug‐associated dysphoria, which self‐resolved.

### Duration of surgery and hospitalisation

The median duration of surgery was 152 minutes (range: 80–210 minutes). The median duration of hospitalisation was 2 days (range: 1–4 days) (Table 1).

### Postoperative CT imaging findings

Three dogs received a postoperative CT immediately following surgery, which revealed subcutaneous emphysema of the ventral neck from surgery as well as a defect created in the ventral bulla, confirming appropriate anatomical identification.

### Histopathology of the bulla

Histopathology of the affected bulla was obtained in seven of eight dogs. In the remaining dog where histopathology was not performed, a postoperative CT was performed, which confirmed that there was an osteotomy of the bulla. All samples were interpreted as suppurative lymphoplasmacytic inflammation suggestive of otitis media. Two of the seven samples had concurrent inflammatory polyps, one sample with granulation tissue and one sample with tympanokeratoma/cholesteatoma (Table 2).

**TABLE 2:** Intra‐ and postoperative complications, histopathology and culture results in eight French bulldogs that underwent a fluoroscopic guided ventral bulla osteotomy.

		Postoperative complications				
Dog	Intraoperative complications	<14 days	>14‐day follow‐up duration (days)	Description	Histopathological diagnosis	Culture and sensitivity results
1	None	Static left head tilt and facial nerve paralysis	120	Static left head tilt and facial nerve paralysis, chronically on steroids due to recurring otitis media, total ear canal ablation surgery was recommended.	Lymphoplasmacytic and neutrophilic otitis media with fibroplasia	No growth
2	None	Static right head tilt	440	Static right head tilt	Granuloma tissue	No growth
3	None	Decreased left palpebral response	726	Static decreased left palpebral response	Chronic‐active otitis media with inflammatory polyp	No growth
4	None	Mild vestibular ataxia	210	Mild but improved vestibular ataxia	Tympanokeratoma/cholesteatoma	Methicillin‐resistant *Staphylococcus pseudintermedius*
5	None	Static left head tilt and mild positional nystagmus	180	Static left head tilt	Chronic suppurative lymphoplasmacytic otitis media with inflammatory polyp	*S. pseudintermedius*
6	None	Static left head tilt and mild vestibular ataxia	730	Static left head tilt, resolved vestibular ataxia	Suppurative haemorrhagic otitis media with granulation tissue	*S. pseudintermedius*
7	Mild drug‐associated dysphoria, self‐resolved	Static right head tilt	1491	Static right head tilt	Not applicable	Beta‐haemolytic *Streptococcus*
8	None	Static right head tilt	208	Static right head tilt	Suppurative lymphoplasmacytic otitis media	No growth

### Culture and sensitivity testing of the bulla samples

Intraoperative aerobic cultures were performed in all eight dogs, in which four of eight were positive for growth and remaining four negative for growth (Table 2).

### Postoperative follow‐up

The median follow‐up duration was 325 days postoperatively (range: 120–1491 days). At the 14‐day postoperative recheck, six of eight dogs displayed a persistent head tilt, which was static to their preoperative status. None of these dogs showed improvement at their secondary follow‐up. One dog exhibited a decreased palpebral response at the 14‐day postoperative recheck, which was static at 729 days postoperatively.

Two out of five dogs that displayed evidence of secondary meningitis on MRI and CSF diagnostic testing exhibited residual ataxia at the 14‐day recheck. One of these dogs was reported to show signs of improvement at the 210‐day postoperative recheck and the other was found to have resolved signs 2 years postoperatively (Table 2).

One dog developed recurring otitis media in the affected ear 4 months postoperatively. The dog was chronically managed with prednisone and antibiotics by its primary referring veterinarian, with no significant improvement in clinical signs. A TECA‐LBO was recommended as a salvage procedure to eliminate the source of recurring infection but was declined by the owner.

## DISCUSSION

This case series described the technique and outcomes of using intraoperative fluoroscopy to aid the surgical approach to the French bulldog bulla. The current described surgical approach for VBOs in dogs and cats relies heavily on palpation of the bulla and bony landmarks such as the ramus of the mandible.^14^ In a comparative study,^15^ French bulldogs and pugs exhibited more anatomical overlap of their temporomandibular joints and tympanic bullae than mesaticephalic dogs, with the bulla located directly medial to the temporomandibular joint. Anecdotally, distinguishing between the ventral surface of the tympanic bulla and the ventral aspect of the ramus of the mandible is extremely challenging on digital palpation in brachycephalic dogs due to this overlapping of surgical landmarks. In this case series, it was found that the distance between the skin and the bulla was a median of 56.6 mm, preventing palpation of the bulla easily. Due to the inability to palpate the normal anatomic landmarks, fluoroscopy allowed increased confidence in the dissection and identification of the bulla.

Other reported techniques for identification of the bulla for VBO, such as optic image‐guided and transoral VBOs, have their own limitations.^8^, ^16^ Optic image‐guided systems are not readily accessible in veterinary medicine and were only evaluated in dolichocephalic breeds in which identification of the bulla may be more feasible. The study describing a minimally invasive transoral approach for VBOs was performed in 12 cadavers, where only three of 12 dogs were brachycephalic and none were French bulldogs.^16^ The clinical application of the transoral technique has not been reported, and the effectiveness of this approach has not been assessed in live dogs. Given that fluoroscopy is commonly available in referral veterinary hospital settings, the authors believe that fluoroscopic guided VBO can be of added value due to its accessibility and simplicity of the approach.

To the best of the authors' knowledge, there are no previous reports of surgical times for VBOs in brachycephalic dogs. The surgical times reported for the limited number of cases here were comparable to what has been previously reported for TECA‐LBOs in brachycephalic dogs.^17^ Fluoroscopic guidance was introduced in the hope of accelerating bulla identification. With increased comfort with the technique, it is suspected that surgical times will improve, which in turn will increase patient safety.

Despite brachycephalic dogs being reported to have narrower ear canals compared to dolichocephalic dogs, VBO should still be considered as an alternative treatment option for otitis media, because the procedure is less invasive than TECA‐LBO and residual hearing can potentially be preserved.^5^, ^11^, ^18^ In addition, improvement in preoperative clinical signs was documented in seven of eight dogs in our study without resorting to TECA‐LBO, which is considered a salvage procedure.^19^ Even though VBO did not consistently resolve preoperative clinical signs such as head tilt, vestibular ataxia and facial nerve paralysis, these clinical signs were reported to be mild, and long‐term improvement of ataxia and facial nerve paralysis was observed.

Limitations of this study included the small sample size in this descriptive study. With no control group and standardised outcome measures, no statements can be made regarding the technique's superiority to any other described technique for bulla identification.

In conclusion, intraoperative use of fluoroscopy could be considered to guide the identification of the bulla in VBOs in brachycephalic breeds, where the surgical approach can be challenging due to their skull conformation.

## AUTHOR CONTRIBUTIONS

**Hui Yu Lu**: Data curation (lead); writing—original draft (lead); writing—review and editing (lead). **Jeffery J. Biskup** and **Lea R. Mehrkens**: Conceptualisation (supporting); methodology (equal); writing—review and editing (supporting). **Debbie Reynolds**: Conceptualisation (lead); methodology (lead); writing—review and editing (lead).

## CONFLICTS OF INTEREST

The authors declare they have no conflicts of interest.

## FUNDING INFORMATION

The authors received no specific funding for this work.

## ETHICS STATEMENT

The authors confirm that the ethical policies of the journal, as noted on the journal's author guidelines page, have been adhered to. No formal ethical approval was required because this was a series of clinical cases.

## Data Availability

The data presented in this study are available on request from the corresponding author due to privacy reasons.
